# Easy Test for Visceral Leishmaniasis and Post–Kala-azar Dermal Leishmaniasis

**DOI:** 10.3201/eid1707.100801

**Published:** 2011-07

**Authors:** Samiran Saha, Ramaprasad Goswami, Netai Pramanik, Subhasis K. Guha, Bibhuti Saha, Mehebubar Rahman, Sudeshna Mallick, Dolanchampa Modak, Fernando O. Silva, Ivete L. Mendonca, Dorcas L. Costa, Carlos H. N. Costa, Nahid Ali

**Affiliations:** Author affiliations: Indian Institute of Chemical Biology, Kolkata, India (S. Saha, N. Ali);; School of Tropical Medicine, Kolkata (R. Goswami, N. Pramanik, S.K. Guha, B. Saha, M. Rahman, S. Mallick, D. Modak);; Federal University of Piauí, Teresina, Brazil (F.O. Silva, I.L. Mendonca, D.L. Costa, C.H.N. Costa)

**Keywords:** Leishmania, visceral leishmaniasis, ELISA, dipstick, parasites, letter

**To the Editor:** Diagnosis of visceral leishmaniasis (VL), fatal if untreated, is complex because the symptoms are the same for many fever-associated ailments. Despite limitations, diagnosis remains based on finding *Leishmania* amastigotes in spleen and/or bone marrow aspirates (*1*). Sophisticated laboratory methods, although sensitive, are costly. The immunochromatographic strip test that uses recombinant K39 antigen (rK39), although satisfactory in India, is less sensitive in Africa, Latin America, and Mediterranean regions (*2*). Post–kala-azar dermal leishmaniasis (PKDL), a sequel to VL in India and Africa, is often confused with other skin diseases (*3*,*4*). Diagnosis of VL in dogs in Latin America and Mediterranean countries remains confusing because of rampant asymptomatic infections and elevated antibodies against *Leishmania* spp (*5*).

Earlier we reported the diagnostic potential of *L. donovani* (MHOM/IN/83/AG83) promastigote membrane antigens (LAg) (*3*,*6*). Here we report applicability of LAg-based ELISA and dipstick systems even at primary health centers. Using randomized sampling, we tested samples from 122 kala-azar patients from India, 20 PKDL patients from India, and 40 VL patients from Brazil. VL was confirmed by finding parasites in aspirates. Serum samples were collected before chemotherapy was given. PKDL was diagnosed as described (*3*). Control samples were collected from 24 healthy persons from non–disease-endemic areas in India; 15 healthy persons from disease-endemic areas in India; 20 healthy persons from disease-endemic areas in Brazil; and 21 persons with Hansen disease, 7 with filariasis, 4 with tuberculosis, 1 with lymphoma, 1 with leukemia, 2 with virus-induced fever, and 5 with malaria. Consent was obtained from all human donors. This study was approved by Ethical Committee on Human Subjects at Indian Institute of Chemical Biology and the Ethical Board for Human Subjects and Animal Experimentation of the Federal University of Piauí.

We developed a diagnostic ELISA with modifications of our previous method (*6*). Microtiter plates were coated with 2.5 μg LAg at pH 7.5 (100 μL/well) and kept at 4°C overnight, after which they were blocked with 1% bovine serum albumin, dried, and stored at 4°C as precoated plates. The assay performed at room temperature took ≈2.5 h. Test and control serum samples (1:1,000 dilution, 100 μL/well) were applied to the plates for 45 min and shaken occasionally. Horseradish peroxidase–conjugated goat anti-human immunoglobulin (Ig) G (Genei, Bangalore, India) was applied at 1:5,000 (100 μL/well) for 45 min. Color development with ortho-phenylenediamine (Sigma-Aldrich, St. Louis, MO, USA) was allowed for 5–10 min. Positive results were determined by comparing colors with those on a card previously prepared for positive and negative wells. ELISA, performed for the VL and PKDL patients from India, was 100% sensitive (percentage of patients with confirmed disease and positive test results) and 96.3% specific (percentage of negative controls with negative test results) (Figure, panel A); sensitivity and specificity were higher than that reported earlier (*6*) and by other studies that used crude leishmanial antigens (*2*). One sample from each of filariasis, lymphoma, and disease-endemic area controls was marginally false positive. Seropositivity was diagnosed for 1 patient who had a negative spleen aspirate but clinical signs of VL and for 1 patient who refused spleen or bone marrow aspiration.

**Figure:**
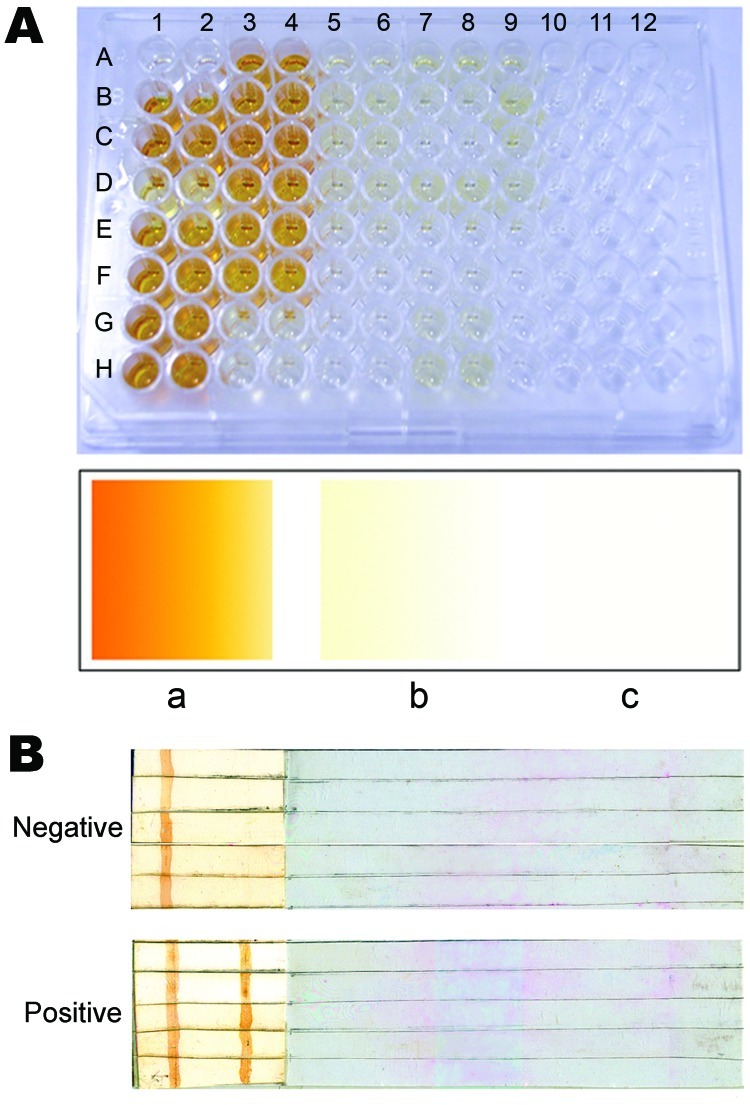
Representative results of ELISA and dipstick testing. A) Samples underwent ELISA in duplicate. Upper panel, positive samples in duplicate (1–2 and 3–4) in wells A–H, except A1–A2 (blank), and G3–G4 and H3–H4 (negative controls). Wells in columns 5–10 represent different negative controls in duplicate (5–6, 7–8, and 9–10), except F9–F10, G9–G10, H9–H10, and all wells in columns 11–12 (unused wells). Lower panel, the reference color card: a, positive; b, negative; c, blank. B) Dipstick test results. The left color band is the internal control line; the right color band is the test line.

To avoid any visible cross-reaction in the dipstick assay, we optimized LAg concentration, test serum dilution, and control serum dilution. Optimum concentration for human studies is 500 μg/mL LAg, 1:2,000 serum dilutions, 1:2,000 horseradish peroxidase–conjugated goat anti-human IgG, and 15 mg 3,3′-diaminobenzidine (Sigma-Aldrich) as substrate in 30 mL Tris-buffered saline. LAg was bound to a long nitrocellulose piece at the test line (line on which LAg is coated). Goat anti-human IgG (Genei) at 1:25 was coated as an internal control line. Free sites were blocked with 2% bovine serum albumin containing 0.01% NaN_3_ and were air dried. LAg-coated membranes were affixed to the end of a plastic support (with a free end as handle) with double-adhesive tape, cut into 4-mm–wide sticks, and stored at room temperature. During the testing process at room temperature, dipsticks were incubated in diluted serum for 30 min, washed 2×, incubated for 30 min with secondary antibody, washed 3×, and incubated in substrate solution for 1 min. Finally, dipsticks were washed in water, dried on tissue paper, and examined for specific reaction. When stored at room temperature without desiccation, dipsticks performed consistently for 12 months. Dipsticks appeared equally sensitive and specific (100%) for VL from India and Brazil and for PKDL. Because internal control lines remained positive, analyses were considered valid (Figure, panel B).

LAg dipsticks are more sensitive for diagnosing VL in Brazil than rK39 (*7*) and cost ≈70× less (*8*). Although further validation with a larger sample size and healthy controls from disease-endemic areas and controls for other diseases is warranted, these easy, simple, and low-cost methods could emerge as efficient tools for diagnosis of VL and PKDL.

## References

[1] da Silva MR, Stewart JM, Costa CH. Sensitivity of bone marrow aspirates in the diagnosis of visceral leishmaniasis. Am J Trop Med Hyg. 2005;72:811–4. 15964968

[2] Saha S, Mondal S, Banerjee A, Ghose J, Bhowmick S, Ali N. Immune responses in kala-azar. Indian J Med Res. 2006;123:245–66. 16778308

[3] Saha S, Mazumdar T, Anam K, Ravindran R, Bairagi B, Saha B, *Leishmania* promastigote membrane antigen-based enzyme-linked immunosorbent assay and immunoblotting for differential diagnosis of Indian post–kala-azar dermal leishmaniasis. J Clin Microbiol. 2005;43:1269–77. 10.1128/JCM.43.3.1269-1277.2005 15750095 PMC1081224

[4] Zijlstra EE, Khalil EA, Kager PA, El-Hassan AM. Post–kala-azar dermal leishmaniasis in the Sudan: clinical presentation and differential diagnosis. Br J Dermatol. 2000;143:136–43. 10.1046/j.1365-2133.2000.03603.x 10886148

[5] Romero GAS, Boeleart M. Control of visceral leishmaniasis in Latin America—a systematic review. PLoS Negl Trop Dis. 2010;4:e584. 10.1371/journal.pntd.0000584 20098726 PMC2808217

[6] Anam K, Afrin F, Banerjee D, Pramanik N, Guha SK, Goswami RP, Immunoglobulin subclass distribution and diagnostic value of *Leishmania donovani* antigen–specific immunoglobulin G3 in Indian kala-azar patients. Clin Diagn Lab Immunol. 1999;6:231–5. 10066659 10.1128/cdli.6.2.231-235.1999 PMC95692

[7] Carvalho SF, Lemos EM, Corey R, Dietze R. Performance of recombinant K39 antigen in the diagnosis of Brazilian visceral leishmaniasis. Am J Trop Med Hyg. 2003;68:321–4. 12685638

[8] Sundar S, Maurya R, Singh RK, Bharti K, Chakravarty J, Parekh A, Rapid, noninvasive diagnosis of visceral leishmaniasis in India: comparison of two immunochromatographic strip tests for detection of anti-K39 antibody. J Clin Microbiol. 2006;44:251–3. 10.1128/JCM.44.1.251-253.2006 16390983 PMC1351954

